# An emerging role for long non-coding RNAs in cancer metastasis

**DOI:** 10.3389/fgene.2014.00234

**Published:** 2014-07-18

**Authors:** Jason T. Serviss, Per Johnsson, Dan Grandér

**Affiliations:** Grander Lab, Department of Oncology and Pathology, Karolinska InstitutetStockholm, Sweden

**Keywords:** lncRNA, non-coding RNA, metastasis, cancer, epithelial-mesenchymal-transition

## Abstract

Metastasis is a multistep process beginning with the dissemination of tumor cells from a primary site and leading to secondary tumor development in an anatomically distant location. Although significant progress has been made in understanding the molecular characteristics of metastasis, many questions remain regarding the intracellular mechanisms governing transition through the various metastatic stages. Long non-coding RNAs (lncRNAs) are capable of modulating both transcriptional and post-transcriptional regulation, and thus, coordinating a wide array of diverse cellular processes. Current evidence indicates that lncRNAs may also play a crucial role in the metastatic process through regulation of metastatic signaling cascades as well as interaction with specific metastatic factors. Here we summarize a subset of lncRNAs with proposed roles in metastasis and, when applicable, highlight the mechanism by which they function.

## INTRODUCTION

The recent discovery that ~75% of the human genome is transcribed to RNA, with only ~1.2% being responsible for protein coding, indicates that a large portion of the genome is dedicated to regulating a relatively small amount of effectors (Kapranov et al., 2002; International-Human-Genome-Sequencing-Consortium, 2004; Carninci et al., 2005; Katayama et al., 2005; Djebali et al., 2012). Among the newly discovered RNA elements, long non-coding RNAs (lncRNAs) have been identified to have functional roles in a diverse range of cellular functions such as development, differentiation, and cell fate as well as disease pathogenesis (Rinn et al., 2007; Guan et al., 2013; Kung et al., 2013; Lee and Bartolomei, 2013). lncRNAs are generally defined as RNA transcripts longer than 200 nt with no coding potential as indicated by lack of a discernable open reading frame. lncRNAs typically exhibit more tissue specificity, lower expression levels, and less conservation than protein coding transcripts (Derrien et al., 2012). Although thousands of lncRNAs have been identified, their function and involvement in disease remains poorly studied. Intriguingly, lncRNAs have moved into the limelight within cancer research where their expression has been shown to be dysregulated in multiple cancer types and examples of lncRNA-mediated regulation of several tumorigenic factors has been demonstrated (Morris et al., 2008; Yap et al., 2010; Brunner et al., 2012; Johnsson et al., 2013). Although studies examining the role of lncRNAs in specific oncogenic processes are limited to date, emerging evidence suggests them to have essential roles in regulation of the metastatic process.

## METASTASIS

Metastatic disease frequently represents an incurable impasse and a dim prognosis for patients receiving this diagnosis. The mechanisms regulating metastatic progression have been largely unknown hindering the development of novel treatment options. In recent years a renewed focus on underlying molecular dynamics and cellular pathways prompting metastatic transition has begun to shed light on this previously scantly explored territory.

Metastasis is commonly viewed as a linear chain of events resulting in the relocation of tumorigenic cells from the primary tumor site to a distant location. The stages of metastasis may be divided into the following categories: (1) invasion/intravasation, the escape of cells from the primary tumor into the blood or lymphatic system, (2) survival and arrest, the avoidance of apoptosis and shear stress in the vasculature leading to arrest at a secondary site, (3) extravasation, infiltration into the parenchyma of the distant tissue, (4) micrometastasis, survival within a new microenvironment, and (5) metastatic colonization, initiation of proliferative capability and growth. Despite the fact that these steps represent an oversimplification of the actual events, they serve as a useful model to support our understanding of the biological events occurring during metastasis.

The activation of specific cell autonomous pathways has been associated with the acquisition of necessary metastases-promoting attributes such as increased migration, altered fate specification, independence of cell–cell communication, avoidance of apoptosis, and transient quiescence. Wingless (WNT) signaling, resulting in the nuclear translocation of β-catenin and activation of its downstream transcriptional targets, as well as transforming growth beta (TGF-β) signaling are two more classically recognized pro-metastatic pathways although the NOTCH, Akt-mTOR, JNK, and Hedgehog (Hg) pathways have all been associated with procurement of various metastatic features (Jiao et al., 2008; Dubrovska et al., 2009; Polyak and Weinberg, 2009).

The developmental programs, epithelial-mesenchymal- transition (EMT) and its inverse process, mesenchymal-epithelial-transition (MET), are also partly defined mechanisms which metastatic cells undergo to navigate transitional barriers within the metastatic process (for a detailed review please refer to Brabletz, 2012). EMT is typically thought to be primarily involved in the invasion and intravasation stages where events such as downregulation of the adhesion molecule E-cadherin and upregulation of the mesenchymal marker vimentin lead to increased mobility capacity. Many of the pathways known to be involved in metastasis also have close connections with EMT. Notably, WNT and TGF-β contribute to the EMT activation network via β-catenin-mediated activation of EMT inducing factors and SMAD protein interactions, respectively (Padua and Massague, 2009). Hypoxic conditions in tumor cores are also conducive to the induction of EMT via upregulation of HIF-1α (hypoxia-inducible factor-1α), HGF/SF (hepatocyte growth factor/scatter factor), and other known pro-EMT factors (Gort et al., 2008).

Beginning with the initial observation that snail family zinc finger (SNAIL) 1 associates with the E-cadherin promoter a host of other transcription factors have been subsequently identified to regulate EMT (Batlle et al., 2000). Notably SNAIL2, Zeb1 and Zeb2 (zinc finger E-box binding homeobox), Twist1 (twist basic helix-loop-helix transcription factor 1), and PRRX1 (paired related homeobox 1) have all been shown to be important agonistic EMT factors (Ocana et al., 2012; De Craene and Berx, 2013). Importantly, a network of interactions is necessary to tip the balance and initiate the EMT program. Furthermore, alternative splicing, post-transcriptional regulation, and microRNAs (miRNA) have all been shown to be important mechanisms in the regulation of EMT (reviewed in De Craene and Berx, 2013). Despite the accumulating information concerning the molecular mechanisms underlying EMT, relatively little is known regarding MET regulation. Disseminating tumor cells which undergo EMT become quiescent, inhibiting their ability to form macrometastases. MET is one of the proposed transitions that disseminated cells may utilize to re-differentiate, thus recapturing their proliferative capability. Several lines of evidence indicate that the ability of disseminated cells to undergo MET, may represent the rate-limiting step in the metastatic process underling the need for continued research in this area (reviewed in Brabletz, 2012).

Here we review individual lncRNAs implicated in the metastatic process. We highlight, when possible, the mechanisms by which the lncRNAs function and how they are themselves regulated. We explore several known lncRNAs, which may potentially be involved in metastasis as well as those which have only recently been discovered and provide interesting targets for further future characterization.

## METASTASIS-ASSOCIATED LUNG ADENOCARCINOMA TRANSCRIPT 1

Metastasis-associated lung adenocarcinoma transcript 1 (MALAT1), also known as NEAT2, has been implicated in several studies as having an important role in metastasis. This transcript localizes to a frequent chromosomal translocation breakpoint region, the 11q13 locus, previously recognized to have a role in tumor development and invasion (Bekri et al., 1997; Chakrabarti et al., 1998; Davis et al., 2003). Ji et al. (2003) previously showed that patients with non-small cell lung cell cancer (NSCLC) exhibiting high overexpression of the MALAT1 transcript were five times more likely to have a metastatic event compared to low expressing patients. Since this discovery, investigations have been initiated to characterize the MALAT1 transcript and the mechanisms by which it functions. Under normal conditions MALAT1 is broadly transcribed across a large range of tissue types including pancreas, lung, prostate, colon, and brain (Ji et al., 2003). Post-transcriptional nuclear cleavage of the primary MALAT1 transcript, by RNase P and RNase Z, gives rise to the nuclear retained MALAT1 transcript and MALAT1-associated small cytoplasmic RNA (mascRNA), a 61 nt ncRNA (Wilusz et al., 2008).

Early reports indicated that MALAT1 localizes to nuclear speckles where it interacts with members of the serine/arginine-rich (SR) family of nuclear phosphoproteins and regulates their phosphorylation status (Sanford et al., 2009; Tripathi et al., 2010). SR protein levels and phosphorylation have known roles in the regulation of alternative splicing patterns, indicating that MALAT1 may serve to regulate splicing. Antisense oligonucleotide-mediated knockdown of MALAT1 in human HeLa cells confirmed this hypothesis resulting in modified mRNA splicing of transcripts whose isoforms are regulated by nuclear speckle-associated proteins (Tripathi et al., 2010). Several of these mRNAs code for proteins with known roles in oncogenic and metastatic pathways such as WNT signaling (CAMK2B and HMG2L1), cytoskeletal organization (ARHGEF1) as well as cell cycle, DNA damage, and metabolism (CDK7, B-MYB, SAT1). Collectively, these results implicate MALAT1 in the post-transcriptional modification of genes involved in established processes that are vital to the metastatic cascade. Due to recent results indicating aberrant splicing patterns in various cancer types, it may well prove that MALAT1 dysregulation is partially responsible for this observation (Watson and Egland, 2010; Menghi et al., 2011).

Contrary to this, several other studies have indicated that the involvement of MALAT1 in nuclear speckle-mediated regulation of alternative splicing is limited to non-existent (Nakagawa et al., 2012; Zhang et al., 2012; Gutschner et al., 2013). MALAT1 knockout mice were shown to have normal nuclear speckles as well as unaltered SR protein levels, localization, and phosphorylation status (Nakagawa et al., 2012; Zhang et al., 2012). Continued investigation utilizing MALAT1 knockout human A549 lung adenocarcinoma cells, showed that alternative splicing was not significantly altered compared to control cells (Gutschner et al., 2013). It should be noted that alternative functions, such as sub-nuclear compartmentalization of growth control genes, have been described for MALAT1 and could account for some of this discrepancy (Yang et al., 2011).

Despite the uncertainty surrounding the mechanism of action by which MALAT1 functions, some agreement can be seen in studies investigating its dysregulation in oncogenesis and metastasis. After the original discovery that MALAT1 is overexpressed in NSCLC, several other studies have been published showing the overexpression of MALAT1 in a multitude of cancer types such as colorectal, breast, pancreas, bladder, and prostate cancers (Lin et al., 2007; Han et al., 2013). Studies including patient cohorts exhibiting a metastatic cancer phenotype have noted increased levels of MALAT1 in high-risk metastatic tumors compared to low risk tumors (Schmidt et al., 2011; Ying et al., 2012; Han et al., 2013). Interestingly, gene expression of known regulators of EMT have been shown to be affected by MALAT1 dysregulation. Gutschner et al. (2013) demonstrated the downregulation of the EMT-related proteins LPHN2 and ABCA1, as well as several other metastasis regulators, in the MALAT1 knockout A549 cell line. Using two separate murine xenograft models, the group also confirmed a reduction of lung metastasis with reduced MALAT1 levels. Additional studies performed in the T24 bladder cancer cell line by Ying and coworkers showed that siRNA-mediated MALAT1 knockdown was associated with decreased levels of the EMT related transcription factors Slug, Zeb1, and Zeb2, as well as decreased nuclear localization of β-catenin. The group also noted that E-cadherin expression was increased in this cell line upon MALAT1 siRNA targeting (Ying et al., 2012). Notably, a direct association of MALAT1 and the c-Jun transcription factor, with known roles in TGF-β factor signaling and nuclear import of SMAD proteins, has also been documented (Zhang et al., 1998; Yang et al., 2011).

Collectively, these results indicate that MALAT1 may have an important role in metastatic cancer. Despite the fact that the 61 nucleotide ncRNA, mascRNA, is known to originate from the primary MALAT1 transcript, relatively few studies have addressed the function of this short transcript (Xu et al., 2011). The conflicting results regarding the mechanism of function of MALAT1 could be accounted for in several ways. It is possible that MALAT1 functions via interaction with specific proteins to achieve different end points dependent on cellular context. Another possibility is MALAT1s role in regulating specific cellular processes may be redundant due to shared regulatory mechanisms via other cell type specific mediators. Regardless of this, the emerging role of MALAT1 and mascRNA in oncogenesis and metastasis warrants continued research.

## H19

The oncofetal H19 gene was the first imprinted ncRNA to be identified, and the H19/IGF2 (insulin-like growth factor 2) locus has long served as a model for genomic imprinting. High levels of H19 expression are typically only seen during embryonic development and, with the exception of muscle and cardiac tissue, H19 is strongly downregulated after birth (Pachnis et al., 1988; Poirier et al., 1991). Loss of imprinting (LOI) at the 11p15.5 H19/IGF2 locus gives rise to an imbalanced expression of H19 and IGF2, the clinical features of which are seen in Beckwith–Wiedemann syndrome (Sparago et al., 2004). Patients suffering from this syndrome exhibit postnatal overgrowth and increased risk for childhood cancers, such as Wilms’ tumor (DeBaun and Tucker, 1998). H19 dysregulation has also been implicated in a variety of other cancers such as colorectal cancer (Tsang et al., 2010), hepatocellular carcinoma (HCC; Ariel et al., 1998), breast cancer (Lottin et al., 2002), and bladder cancer (Luo et al., 2013), among others.

**FIGURE 1:**
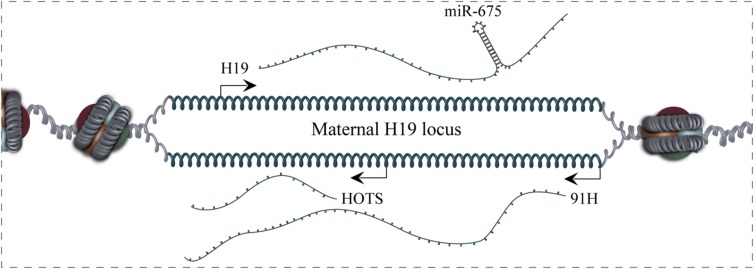
**Genomic organization of the maternal H19 locus.** H19, H19 opposite tumor suppressor (HOTS), miR-675, and 91H are expressed from the H19/IGF2 locus. HOTS is a protein coding gene transcribed antisense to H19. H19 is also the host gene for the miR-675, which is a prognostic marker in several cancer types. 91H is a 120-kb nuclear localized H19-antisense transcript, which is overexpressed in breast cancer tissues.

The H19 locus is host to a multitude of maternally imprinted coding and non-coding transcripts including H19, miR-675, H19 opposite tumor suppressor (HOTS), and 91H (**Figure 1**). Several of these transcripts have also been implicated in the oncogenic process although in depth characterization is lacking for many of them (Wilkin et al., 2000; Berteaux et al., 2008; Onyango and Feinberg, 2011; Schmitz et al., 2011; Schultz et al., 2012). The amount of transcripts arising from the H19 locus has, in many cases, complicated elucidation of the function of H19, not in the least from studies performed previous to the other transcripts discovery.

Several direct lines of evidence indicate that H19 is involved in the metastatic process with its role being best characterized in bladder and breast cancer. Using the T24P bladder carcinoma cell line, Ayesh et al. (2002) overexpressed H19 and thereafter used expression array analysis to identify differentially expressed genes. The group identified altered levels of known metastatic and invasive phenotype regulators and concluded that H19 regulates genes involved in invasiveness, migration, and angiogenesis. Further characterization of the role of H19 in bladder cancer showed that H19 is commonly overexpressed in primary human tumor samples that subsequently metastasize (Luo et al., 2013). Additionally, H19 was found to associate with enhancer of zeste homolog 2 (EZH2) and to downregulate E-cadherin as well as regulating WNT signaling via inhibition of the WNT-signaling antagonist Nkd1 (naked cuticle homolog 1). Contrary to this, detailed studies of human bladder carcinoma samples indicate that H19 levels decrease with increasing tumor grade (Ariel et al., 2000). This could indicate that H19 is involved at early time points in the invasion process in bladder carcinoma, such as in the response to hypoxia or EMT. In fact, H19 has been reported to be strongly induced by hypoxic conditions, potentially via a HIF-1α-mediated mechanism (Matouk et al., 2007).

In accordance with studies examining bladder carcinoma, several investigations have also implicated H19 in breast cancer metastasis, where its expression in epithelial cells localized at the epithelial/mesenchymal boundary has been linked to a poor prognosis (Dugimont et al., 1995). Furthermore, *in vitro* characterization showed that HGF/SF was capable of causing increased expression of H19 in epithelial cells, leading to a subsequent cell morphology indicative of increased cell motility (Adriaenssens et al., 2002). Using an elegant mouse mammary tumor model, allowing differentiation between individual stages in the metastatic cascade, Yang et al. (2004) identified H19 as highly upregulated, not only in initial metastatic stages, but also throughout the entire metastatic process. An additional study seeking to link several breast cancer predisposing single nucleotide polymorphisms (SNPs) to clinical characteristics and prognosis, found that carrying the homozygous risk allele for the rs2107425 SNP, located in intron 1 of H19, was significantly associated with short metastatic free survival (Riaz et al., 2012). Upon analysis of H19 expression from these patients it was found that this SNP did not affect H19 expression, suggesting that this genotype may either alter effective splicing of H19 or potentially the expression of one of the H19 antisense transcripts. Demonstrations of lncRNA SNPs resulting in an altered expression of lncRNA-regulated transcripts has, in fact, been reported (Ling et al., 2013).

H19 expression in hepatic metastases arising from 9 different primary tumor types has also been evaluated (Fellig et al., 2005). 80% of these hepatic metastases were shown to exhibit H19 expression, with over half of them being classified as having high expression. Furthermore, H19 expression has been correlated with tumor invasion in the reproductive organs and neoplastic cell invasion of the myometrium (Lottin et al., 2005).

This growing body of evidence indicates that H19 has a distinct pro-metastatic role. Some studies provide evidence refuting this hypothesis indicating that H19 is, in fact a negative regulator of metastasis (Zhang et al., 2013), while other lines of evidence indicate H19s role to be primarily in regulating growth. Reconciliation of these findings may be provided by the hypothesis that H19 arbitrates diverse functions in different cancer types or at unique stages of metastasis. Due to its possible role in events early in the metastatic cascade, such as hypoxia induced invasion, regulation of EMT-related processes and mediation of epithelial/stromal communication leading to cell morphogenesis, it may also be hypothesized that H19 is involved in acquiring an early invasive phenotype. The increasing amount of information regarding the complex regulation of the H19/IGF2 locus and the functional gene products arising from here will, no doubt, in the future lead to a deeper understanding of its role in metastasis.

## HOX ANTISENSE INTERGENIC RNA

The HOX loci have long been known to host an abundance of ncRNAs whose function was poorly understood. In an attempt to identify these ncRNAs, Rinn et al. (2007) created an ultrahigh-resolution tiling microarray and detected 407 discrete transcribed regions within four HOX loci. Of these, HOX antisense intergenic RNA (HOTAIR) was found to be transcribed antisense to the HOXC locus, and preferentially expressed in posterior and distal sites along the developmental axis. Functional studies revealed that, despite its genomic location in relation to HOXC, HOTAIR has little effect on the regulation of its sense transcript. Instead, HOTAIR was shown to function in *trans* to negatively regulate HOXD via increased Polycomb repressive complex 2 (PRC2) occupancy at the HOXD locus. Subsequent studies found that the regulatory dominion of HOTAIR is not exclusive to the HOXD locus (Chu et al., 2011). In fact, chromatin isolation by RNA purification (ChIRP) allowed for the discovery of 832 HOTAIR genomic occupancy sites that displayed a high level of co-occupancy with PRC2 components. These studies also utilized PRC2 deficient cells showing HOTAIR occupancy to be largely unchanged indicating its role in the recruitment of PRC2 to specific genomic loci in a global fashion much as it does locally at the HOXD locus.

**FIGURE 2:**
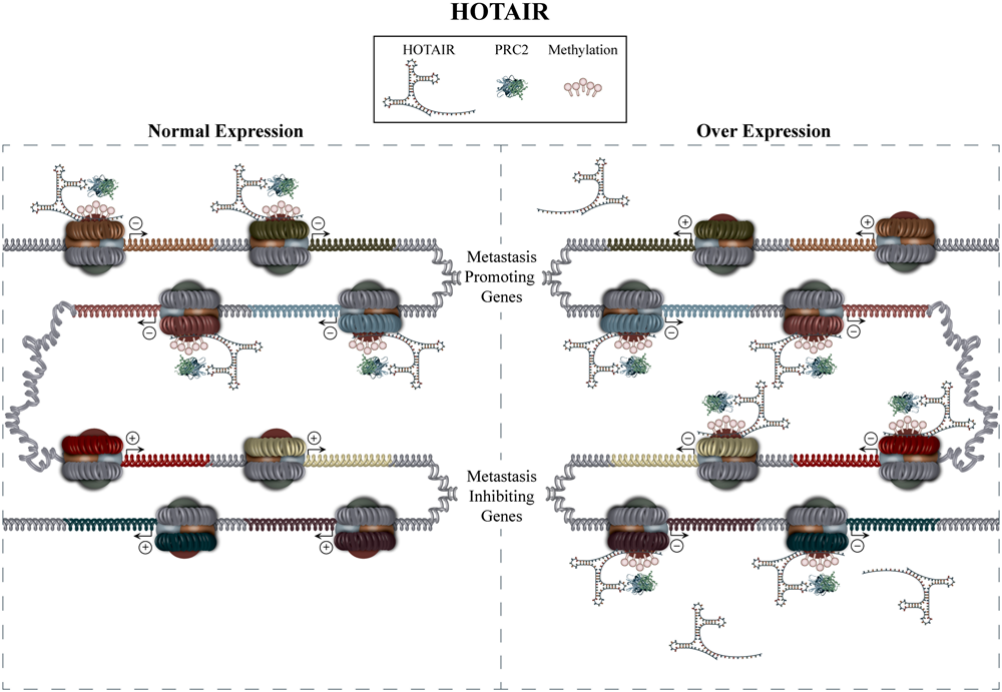
**Hox antisense intergenic RNA mediated regulation.** The recruitment of PRC2 to specific gene sets is mediated by HOTAIR in *trans*, consequently increasing methylation and inactivation of the targeted genes promoters. Overexpression of HOTAIR causes PRC2 to target an alternative gene set, thus mediating a gene expression profile conducive to metastasis. The mechanism by which HOTAIR overexpression mediates de-repression of some genes is, thus far, unknown.

HOTAIR role in metastasis has been confirmed in several cancer types including breast, gastrointestinal stromal tumors, HCC, and non-small cell lung cancer. Initially, a study by Gupta et al. (2010) showed HOTAIR to be overexpressed up to 2000 fold in breast cancer metastases, with its expression being a significant predictor of metastasis and death independent of other risk factors such as tumor size, stage, and hormone receptor status. The study went on to show that overexpression of HOTAIR retargets PRC2 to an alternative gene set, which facilitates expression patterns promoting invasion and motility (**Figure 2**). *In vivo* and *in vitro* experiments also supported a causative role for HOTAIR overexpression in the procurement of a pro-metastatic phenotype. HOTAIR levels have also been shown to be increased in primary tumors from patients with HCC, which exhibit lymph node metastasis, as well as having significant association with a shorter 3 year cumulative recurrence-free survival (Geng et al., 2011). Results in this latter study also indicate that HOTAIR may serve to increase the expression of the pro-metastatic factors VEGF and MMP-9. Studies examining HOTAIRs role in non-small cell lung cancer provide evidence that it is overexpressed in patients exhibiting advanced stage, increased lymph- and vascular invasion, as well as a shorter disease-free survival (Nakagawa et al., 2013). Increased expression of HOTAIR was also found in brain metastases compared to primary tumor samples. Finally, Niinuma et al. (2012) showed overexpression of HOTAIR to be strongly associated with metastasis and poor overall survival in patients with gastrointestinal stromal tumors. This study also showed that 144 of the previously identified HOTAIR target genes exhibit reduced expression in patient samples showing high levels of HOTAIR expression. In addition to its more well known mechanism of action by interacting with PRC2, HOTAIR has also been shown to function as a competitive endogenous RNA (ceRNA). ceRNAs can be non-coding or coding RNAs, that compete with other RNA transcripts for miRNA binding through shared miRNA response elements, thus modulating the pool of miRNAs available for target downregulation. An additional study investigating gastric cancer identified HOTAIR to function as a ceRNA by sequestering miR331-3p (Liu et al., 2014). Decreased miR331-3p levels led to increased HER2 levels whose role in promoting metastasis has been previously identified (Yonemura et al., 1991). Together these studies indicate that therapies capable of returning HOTAIR expression to a baseline level may be beneficial, allowing for the simultaneous return of multiple metastatic genes to normal levels.

## PROSTATE CANCER ASSOCIATED TRANSCRIPT 1

In a cohort of 102 prostate cancer related samples, a genome-wide RNA sequencing (RNAseq) approach was used in order to evaluate differential expression of lncRNAs (Prensner et al., 2011). More specifically, the study utilized prostate tissues including benign adjacent tissues, localized prostate cancers, metastatic tumors and prostate cell lines. RNAseq-based transcript analysis led to the identification of 121 unannotated transcripts, which could accurately discriminate benign, localized and metastatic samples. Further characterization of one of these transcripts, PCAT-1, showed that this lncRNA was upregulated in a subset of high-grade and metastatic cancers. Investigation of putative PCAT-1 regulated genes found 370 genes to be differentially expressed upon siRNA-mediated knockdown of PCAT-1, including subsets with gene ontology annotations such as cell cycle/mitosis, microtubule/cytoskeleton, and microtubule-based processes (Prensner et al., 2011).

Since this initial report, an independent group showed that PCAT-1 may have a similar role in colorectal cancer (Ge et al., 2013). Utilizing a large group of primary patient samples, PCAT-1 was determined to be overexpressed in tumor samples compared to adjacent matched normal tissues. Moreover, increased PCAT-1 expression significantly correlated with distant metastasis as well as short overall survival. The discovery of analogous roles for PCAT-1 in two cancer types may indicate its cancer type independent role as a general regulator of the metastatic phenotype, although further confirmation of this is yet to be provided (Ge et al., 2013).

## lncRNA-LOW EXPRESSION IN TUMOR

lncRNA-low expression in tumor (lncRNA-LET) represents a ncRNA implicated in the suppression of metastasis. Originally discovered in a screen for differentially expressed ncRNAs in hepatitis B virus-related HCC, its expression was further confirmed to be reduced in squamous-cell lung carcinoma and colon carcinoma (Yang et al., 2013a). Clinicopathological characteristic stratification confirmed the relationship between lncRNA-LET expression and micrometastasis as well as the anti-invasive pathological characteristic, encapsulation, in HCC primary human samples. The group’s findings were further substantiated using both tail vein and orthotopic xenograft models with results confirming lncRNA-LET as anti-metastatic. (Yang et al., 2013a).

**FIGURE 3:**
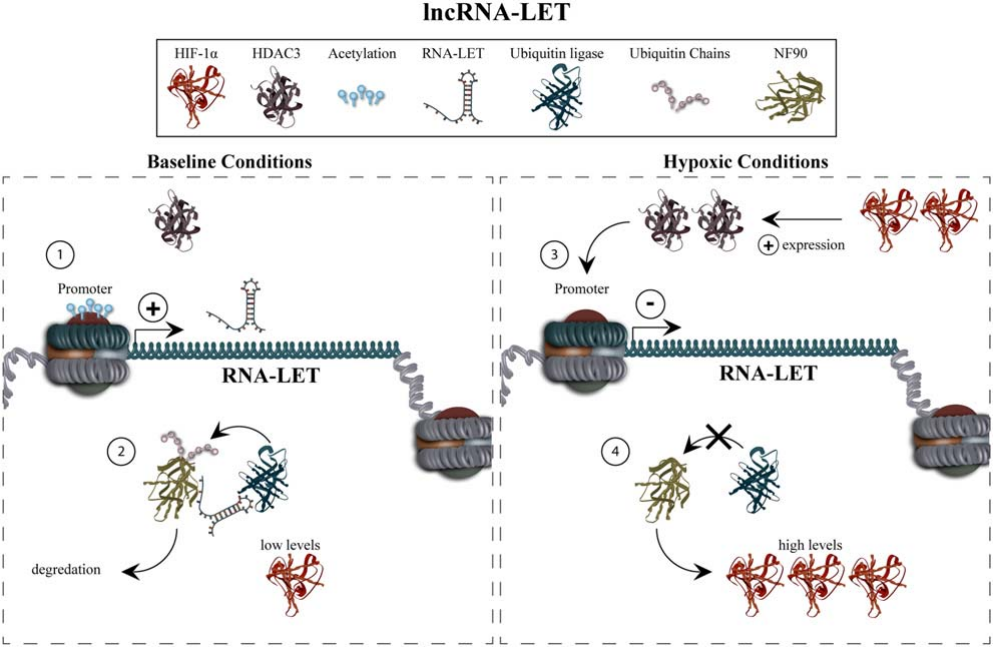
**lncRNA-low expression in tumor regulation and function.** Under normal conditions, lncRNA-LET is expressed (1), resulting in the degradation of NF90 and low HIF -1α levels (2). Hypoxic conditions and concomitant upregulation of HIF -1α increases levels of HDAC3, mediating deacetylation of the lncRNA-LET promoter (3). This results in decreased expression of lncRNA-LET which, in turn, causes decreased ubiquitination of NF90, and thus accumulation of HIF -1α via a positive-feedback mechanism (4). The recruitment of HDAC3, which results in deacetylation of the lncRNA-LET promoter, is currently unexplored.

Mechanistic studies revealed the function of lncRNA-LET to primarily be the regulation of HIF-1α, which has a previously well-documented role in invasiveness and metastasis (**Figure 3**). Hypoxic conditions causing increased levels of HIF-1α resulted in upregulation of HDAC3 (histone deacetylase 3). In turn, this was proposed to lead to reduced lncRNA-LET expression via deacetylation of its promoter. Decreased lncRNA-LET expression gave rise to decreased ubiquitination and thus, accumulation of the NF90 (interleukin enhancer binding factor 3) potentially due to the fact that lncRNA-LET is necessary for interaction between NF90 and a ubiquitin ligase. NF90 was shown to increase HIF-1α levels in a transcription-independent fashion thus indicating that lncRNA-LET is involved in a positive-feedback system promoting HIF-1α levels. These results prompted the group to conclude that lncRNA-LET mediates an anti-invasive phenotype via an indirect reduction of HIF-1α. The group also characterized the ability of lncRNA-LET to regulate the pro-metastatic factor, CDC42, in a non-hypoxia-induced fashion (Yang et al., 2013a).

Analysis of human primary HCC samples strengthened the group’s findings regarding lncRNA-LET’s mechanism of action as well as its underlying importance in metastasis. Specifically, a decreased acetylation status at the lncRNA-LET promoter was found in HCC samples compared to normal adjacent samples. Additionally, an inverse correlation was found for an independent endogenous hypoxia marker, carbonic anhydrase 9 (CA9), and lncRNA-LET expression. Finally, lncRNA-LET and NF90 expression levels were shown to be sufficient to differentiate between HCC staging and the prognostic states of HCC with no tumor thrombus, and HCC with tumor thrombus (Yang et al., 2013a).

## COLON CANCER ASSOCIATED TRANSCRIPT 2

Genome-wide association studies investigating SNPs in cancer have led to the discovery of numerous cancer-associated genomic regions. Investigation of one such SNP, rs6983267, associated with increased risk for colorectal-, prostate-, ovarian-, and inflammatory breast cancer and was found to locate within a highly conserved lncRNA (Ling et al., 2013). This lncRNA, subsequently named colon cancer associated transcript 2 (CCAT2), was shown to have increased expression in metastatic CRC patient tumor samples. The role of CCAT2 in invasion and metastasis was further substantiated using a combination *in vitro* assays and CRC mouse xenograft models showing that CCAT2 overexpression resulted in a higher incidence and greater number of metastatic tumors (Ling et al., 2013).

**FIGURE 4:**
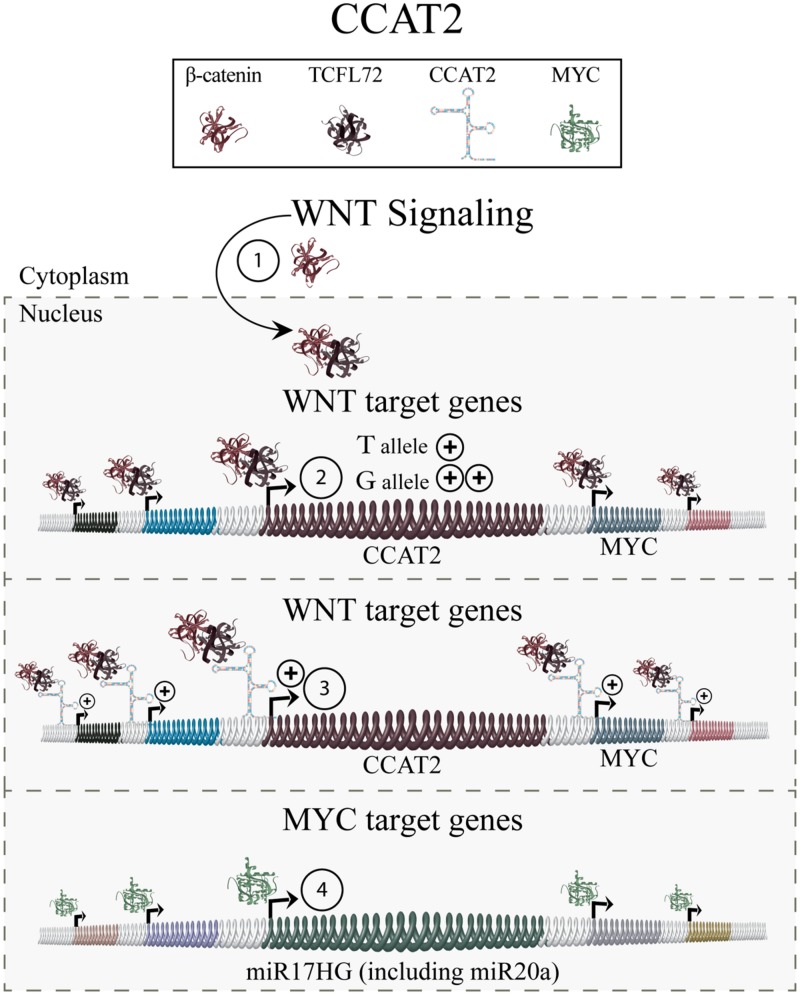
**Colon cancer associated transcript 2.** WNT signaling, cumulating in nuclear translocation of β-catenin and interaction with TCFL72 (1), results in the expression of WNT target genes including CCAT2 (2). Chromosomes harboring the CCAT2 rs6983267 GG allele give rise to increased levels of CCAT2 transcript, and downstream targets such as MYC, compared to the TT allele. CCAT2 then exhibits a positive-feedback reinforcing WNT signaling (3). The increased levels of MYC, in turn, leads to elevated levels of its downstream targets (4) including known metastasis mediators, such as miR17HG and miR20a, and promotes an increased metastatic phenotype.

Continued investigation indicated that CCAT2 is involved in the regulation of WNT-signaling (**Figure 4**). An activation of WNT signaling-induced transcription factor TCF7L2 (transcription factor seven-like 2) was found to increase CCAT2 expression. CCAT2, in turn, modulates expression of WNT target genes, including MYC and thus, its downstream metastasis-associated targets miR17HG and miR20a. Furthermore, CCAT2 overexpressing cell lines showed increased WNT signaling activity, indicating a CCAT2-mediated positive-feedback mechanism on WNT signaling. Collectively, these results indicate that CCAT2 mediates its function by increasing the effects of WNT signaling thus contributing to an enhanced metastatic phenotype (Ling et al., 2013).

Finally, the effect of the rs6983267 allele genotype on CCAT2 levels and function was investigated. Findings indicated that the rs6983267 GG allele results in a significantly higher number of CCAT2 transcripts compared to the TT allele. Moreover, patient samples exhibiting a GG allele and increased CCAT2, also exhibit increased MYC expression, which could not be detected in TT allele patient samples (Ling et al., 2013).

Since the original publication characterizing the CCAT2 transcript in CRC, an additional study examined the prognostic value of CCAT2 in breast cancer (Redis et al., 2013). CCAT2 was found to have increased expression in 2 out of 3 examined primary breast cancer patient sets, although a correlation between the rs6983267 genotype and CCAT expression was not identified. Furthermore, CCAT2 was found to be a valid predictive marker for metastatic-free survival and overall survival in patients with local lymph node metastasis who had received adjuvant CMF (cyclophosphamide, methotrexate, and 5-fluorouracil) therapy. *In vitro* studies in breast cancer cell lines confirmed increased migration capability of CCAT2 overexpressing cells independent of genotype (Redis et al., 2013).

Transcription within the 8q24 region from which CCAT2 arises is complex with an abundance of characterized and uncharacterized transcripts originating here. lncRNAs CCAT1 and CCAT1-L have also been indicated to be involved in the MYC regulation network although their role in metastasis has not been formally investigated (Nissan et al., 2012; Yang et al., 2013b; Xiang et al., 2014). In addition, very lncRNAs arising from this locus encompass several of the CCAT-lncRNAs as well as additional cancer-associated SNPs (Kapranov et al., 2010; St. Laurent et al., 2013). Further investigation of these non-coding transcripts, in conjunction with the functional role that cancer-associated SNPs may play in mediating this function, is necessary to establish their potential role in regulating metastasis.

In summary, these studies provide support for the hypothesis that CCAT2 may have a critical role in invasion and metastasis as well as underlining the utility of genome-wide association studies in identifying potential lncRNAs with disease-associated roles. Furthermore, CCAT2 expression status may prove to be an important predictive marker in CRC, and additionally indicate lymph node positive breast cancer patients that may not benefit from CMF treatment.

## Zeb2/Sip1-NATURAL ANTISENSE TRANSCRIPT

Genome-wide high-throughput sequencing studies have indicated that a large portion of protein coding sense genes also exhibit antisense transcription, known as natural antisense transcripts (NATs; Katayama et al., 2005; Engstrom et al., 2006). These NATs can be both coding and non-coding in nature and may also be co-classified as lncRNAs. The Zeb2 transcription factor is intimately linked with EMT and the loss of an epithelial phenotype (reviewed by Gheldof et al., 2012). Its expression has been linked to advanced carcinoma stages in a variety of cancer types such as breast, ovarian, and gastric cancer (Rosivatz et al., 2002; Elloul et al., 2005). Initial reports characterized a non-coding NAT to the Zeb2 gene with subsequent investigations uncovering the importance of this transcript in the regulation of Zeb2 expression (Nelles et al., 2003; Beltran et al., 2008).

**FIGURE 5:**
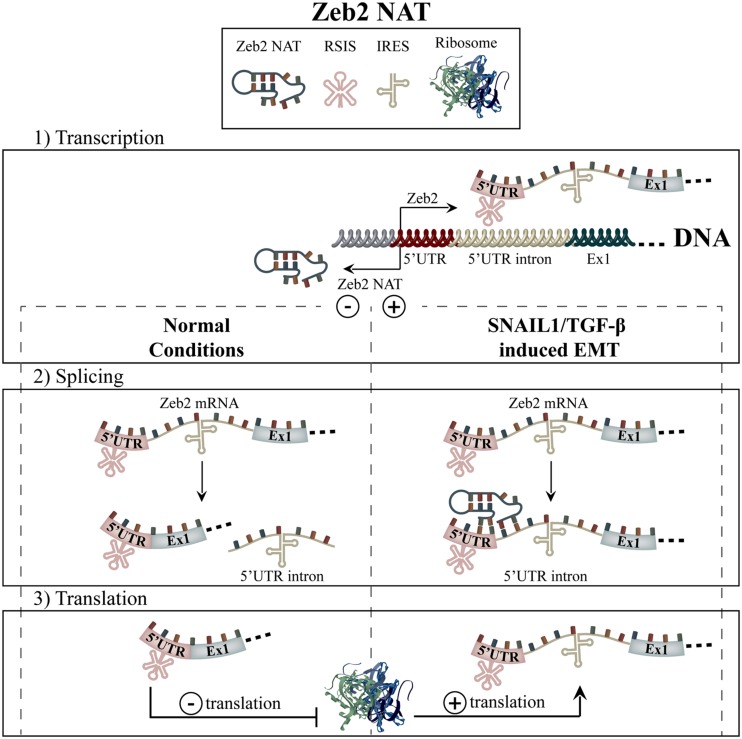
**Zeb2-NAT.** The Zeb2 transcript harbors a ribosome scanning inhibitory sequence (RSIS) in its 5′ UTR, which causes low levels of translation (1). Upon either SNAIL1- or TGF-β-mediated EMT induction, the expression of Zeb2-NAT causes the retention of the Zeb2 transcripts’ 5′ UTR intron by blocking its 5’ splice site (2). An internal ribosome entry sequence (IRES) located in 5′ UTR intron then permits increased ribosomal binding and thus, increased translation of Zeb2 (3).

Under homeostatic conditions, the 5′ UTR of the Zeb2 mRNA transcript contains an inhibitory ribosome scanning sequence, which serves to prohibit its translation (**Figure 5**). Upon induction of SNAIL1 or TGF-β-induced EMT, expression of the Zeb2 NAT is upregulated after which it binds to the 5′ UTR of Zeb2 mRNA (**Figure 4**). This serves to block the 5’-splice site of an internal ribosome entry site (IRES)-containing intron within the Zeb2 mRNA, mediating ribosomal binding and translation. In Zeb2 NAT overexpressing cell lines, the resulting Zeb2 translation was shown to be sufficient to give rise to decreased E-cadherin levels but not to cause full EMT. As well, the intron-retained Zeb2 transcript was shown to have a high inverse correlation with E-cadherin in primary human colon adenocarcinoma samples as well as in breast cancer cell lines (Beltran et al., 2008).

## OTHER LONG NON-CODING RNAs

Several other lncRNAs that have been implicated in metastasis should be mentioned despite the limited amount of information surrounding them. The lncRNA gastric cancer associated transcript 1 (GACAT1), was found to be expressed at lower levels in gastric cancer tissues compared to corresponding normal tissues (Sun et al., 2013b). More importantly, decreased levels of GACAT1 was found to be significantly related to lymphatic and distant metastasis, degree of differentiation, and depth of invasion making it an attractive candidate for mechanistic studies in the future (Sun et al., 2013b).

Another lncRNA, sprouty homolog 4 intronic transcript 1 (SPRTY-IT1), was one of 4 non-coding transcripts found to be abnormally expressed in a screening for differentially expressed lncRNAs in melanoma (Khaitan et al., 2011). This lncRNA is transcribed from the second intron of the sprouty homolog 4 (SPRTY4), and a positive correlation was identified between SPRTY4-IT1 and SPRTY4 expression in several tissues. SiRNA-mediated knock down of the SPRTY4-IT1 transcript produced no effect on the levels of SPRTY4, but *in vitro* assays revealed a decrease of cellular invasion- and mobility capability. Further studies are warranted to ascertain more in depth knowledge concerning the function of this lncRNA in metastasis.

The transcript down-regulated expression by hepatitis B virus X protein (Dreh) was identified as one of 429 dysregulated lncRNAs in the HBx transgenic hepatitis B virus HCC mouse model, when compared to wild type mice (Huang et al., 2013). Of these differentially expressed lncRNAs, Dreh was the only one identified to be consistently downregulated in three different age groups. Xenograft studies indicated that overexpression of Dreh was able to increase tumor differentiation and inhibit metastatic propensity in subcutaneous nude mouse models. Examination of HBV-related human HCC samples found Dreh expression to be significantly correlated with both recurrence free- and overall survival. RNA pull-down experiments revealed vimentin, a common EMT marker and cytoskeletal component of mesenchymal cells, to be associated with lncRNA Dreh. Both overexpression and knockdown studies confirmed Drehs’ ability to negatively regulate cellular levels of vimentin, potentially explaining one mechanism with which it acts to inhibit metastasis (Huang et al., 2013).

The lncRNA-ATB was recently identified to be activated by TGF-β signaling and serve to regulate several metastatic stages in HCC via two separate mechanisms (Yuan et al., 2014). First, lncRNA-ATB was shown to function as a ceRNA, by sequestering members of the miR200 family and thus reducing their availability to target other transcripts. This mediated increased levels of Zeb1/2, resulting in the transition from an epithelial to mesenchymal phenotype and increased metastatic capability. Secondly, and independent of its ceRNA function, lncRNA-ATB was shown to bind to Il-11 mRNA increasing its stability. lncRNA-ATB-mediated increases in Il-11 levels were shown to increase STAT3 activation and promote cellular propensity to successfully survive and colonize distant tissues. lncRNA-ATB expression was shown to be a valid predictor of both recurrence-free- and overall survival in HCC patients and is a promising candidate for further investigation.

Furthermore, Tahira et al. (2011) specifically examined global transcription of lncRNAs in primary and metastatic pancreatic cancer. RNA levels were interrogated in 15 primary adenocarcinoma samples and six distant metastases originating from multiple secondary tumor sites. This revealed 134 ncRNAs to be differentially expressed, the majority (101) being intronic (Tahira et al., 2011).

Finally, several additional antisense transcribed lncRNAs have been implicated as regulators in metastasis. Using a custom microarray, Kohno et al. (2010) defined 256 differentially expressed antisense transcripts comparing primary colorectal tumors to liver metastases samples. Additionally, an antisense transcript to HIF-1α has been shown to be capable of inhibiting HIF-1α during chronic hypoxia, and also being a marker for metastasis free survival in paragangliomas (Uchida et al., 2004; Span et al., 2011). Another example is the LINE-1 chimeric antisense transcript, LCT13, which was shown to induce transcriptional silencing of its sense gene, the anti-metastasis protein TFPI2 (tissue factor pathway inhibitor 2; Cruickshanks et al., 2013). Lastly, the E-cadherin gene, with its well-established role in EMT, has an antisense transcript that has been reported to negatively regulate its protein coding sense gene (Morris et al., 2008). Although, as of yet, further characterization of this transcript’s role in metastasis has not been performed. These antisense ncRNAs all provide potentially interesting candidates for additional studies to allow further understanding of the specific role they may play in metastasis.

## CONCLUSION

The lncRNA-mediated regulation of a diverse range of biological processes is continuously being revealed. Cell cycle regulation, interferon-γ and androgen signaling response, cellular differentiation, and apoptosis are only a few of the thus far reported lncRNA-regulated cellular functions (Rinn et al., 2007; Gomez et al., 2013; Sun et al., 2013a; Takayama et al., 2013). We propose invasion and metastasis to be added to this list due to the ever-growing amount of lncRNAs reported to be differentially expressed in metastatic human samples and lncRNAs’ documented ability to regulate crucial players in the metastatic cascade.

As outlined here, lncRNAs have been shown to play both pro- and anti- metastatic roles via their regulation of hypoxic signaling, the WNT pathway, EMT, and cell adhesion. The role of lncRNAs in other important metastatic features such as cell fate specification, transient quiescence, and avoiding apoptosis are beginning to be uncovered although evidence for dysregulation in human metastatic samples is in some cases lacking (Mourtada-Maarabouni et al., 2008; Hu et al., 2012).

Several studies have analyzed differential expression of lncRNAs in multiple cancer types, comparing normal and tumorigenic samples. Despite this, many of these studies do not include ample patient sample information and/or sample numbers to differentiate between primary and metastasizing tumor lncRNA expression. Identification of lncRNAs mediating progression to specific metastatic stages serves not only to increase our underlying knowledge regarding mechanisms of metastasis but also provides more useful prognostic and diagnostic markers. Ideally, studies aiming to achieve this would divide tumor samples by invasive/metastasizing and secondary tumor, allowing for the identification of lncRNAs, which may be crucial for transitions between these stages of metastasis. As well, new technologies allowing the detection of circulating tumor cells (CTCs; Ramskold et al., 2012) may allow for the interrogation of the circulation survival step in the metastasis cascade. Although this has initially been performed, for example in pancreatic cancer, the use of an endothelial marker (cytokeratin) for CTC detection may exclude cells that have undergone EMT (Yu et al., 2012). Finally, our initial glimpse into the vastness of non-coding RNA transcription was greatly facilitated by the advent of improved whole transcriptome analysis techniques, such as RNA sequencing. The continued development of techniques such as single cell RNA sequencing, direct RNA sequencing, and improved data analysis methods promise to increase our understanding of the non-coding transcriptome and how it is affected in malignancy and metastasis (Ozsolak et al., 2009; Ozsolak and Milos, 2011; Picelli et al., 2014).

Identification of differential expression, while extremely useful, is only the first step in the elucidation of the lncRNA-based molecular mechanisms capable of regulating metastasis. Follow-up with detailed functional studies allows for a deeper understanding of lncRNA regulation and consequently how they regulate their downstream targets. Ultimately, achieving a comprehensive understanding of the complex organization of interaction between both coding and non-coding elements will best facilitate our progress in preventing and treating metastasis. Potentially, future efforts may allow the direct clinical manipulation of lncRNAs involved in metastasis thus aiding in reducing the significant amount of metastasis-related patient mortality.

## Conflict of Interest Statement

The authors declare that the research was conducted in the absence of any commercial or financial relationships that could be construed as a potential conflict of interest.
